# Myenteric Neurons Do Not Replicate in Small Intestine Under Normal Physiological Conditions in Adult Mouse

**DOI:** 10.1016/j.jcmgh.2022.04.001

**Published:** 2022-04-11

**Authors:** Heikki Virtanen, Daniel R. Garton, Jaan-Olle Andressoo

**Affiliations:** 1 Department of Pharmacology, Faculty of Medicine & Helsinki Institute of Life Science, University of Helsinki, Helsinki, Finland; 2 Division of Neurogeriatrics, Department of Neurobiology, Care Sciences and Society (NVS), Karolinska Institutet, Stockholm, Sweden

**Keywords:** ENS, Proliferation, DNA Labelling, IdU, EdU, BrdU, 5-bromo-2'-deoxyuridine, EdU, 5-ethynyl-2'-deoxyuridine, ENS, enteric nervous system, GI, gastrointestinal, IdU, 5-iodo-2'-deoxyuridine, IgG, immunoglobulin G, LM-MP, longitudinal muscle-myenteric plexus, PBS, phosphate-buffered saline

## Abstract

**Background & Aims:**

The enteric nervous system (ENS) is the largest part of the peripheral nervous system; moreover, abnormal ENS development and function are associated with multiple human pathologies. Data from several groups suggest that under normal physiological conditions in adult animals, enteric nerve cells do not replicate. A study by Kulkarni et al in 2017 challenged this view and proposed that nearly 70% of enteric neurons in the myenteric ganglia are born in 1 week. The authors of this study suggested that differences in DNA labelling times and DNA denaturation conditions might explain discrepancies with previous reports. Previous studies were carried out using different conditions and labelling techniques in various regions of the gastrointestinal tract; thus, conclusions have remained elusive.

**Methods:**

Here, we have eliminated those variables by analyzing the whole small intestine using the reagents and conditions that Kulkarni et al used. To exclude variables related to immunohistochemistry, we carried out parallel experiments with “click chemistry”-based detection of DNA replication.

**Results:**

Although proliferation was readily detected in the epithelium, we found no evidence of neuronal replication in the myenteric ganglia.

**Conclusions:**

We conclude that within 1 week under normal physiological conditions, myenteric neurons in the small intestine do not replicate.

## Graphical abstract

**Figure:**
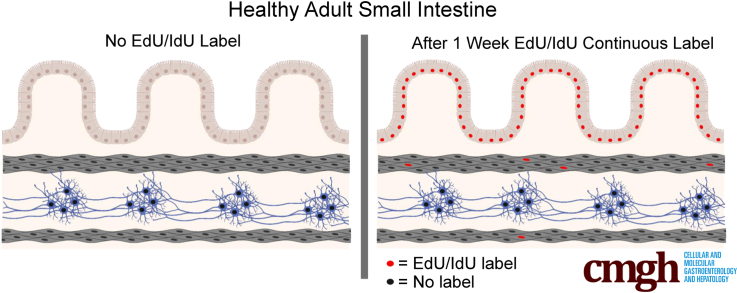

**Summary**

A controversial study by Kulkarni and colleagues has claimed that about 70% of myenteric neurons in the adult small intestine replicate in 1 week. Using the same and alternative methods, we find no evidence of neuronal replication.

The enteric nervous system (ENS) is the largest part of the peripheral nervous system, governing gut motility, secretion, and absorption.^1^ Defects in ENS development are the cause of the most common congenital enteric neuropathy, Hirschsprung’s disease, and mounting evidence suggests that ENS physiology is important in other gastrointestinal (GI) or related pathologies, including inflammatory bowel disease,^1^^,^^2^ colorectal cancer,^3^ and Parkinson’s disease.^1^ Accurate knowledge on ENS biology thus is critical.

Several past studies using various techniques to label sites of DNA replication have suggested that under normal physiological conditions, robust neurogenesis does not occur in the adult gut^4^, ^5^, ^6^, ^7^, ^8^, ^9^, ^10^; however, a recent study by Kulkarni et al^11^ challenged these results and suggested that not only are enteric neurons born in the adult gut, but also almost 70% of them are replaced within 1 week under normal physiological conditions. This is an important conclusion with a possibly far-reaching impact on future research.

First, we reviewed the literature addressing adult enteric neurogenesis under normal physiological conditions in adult mice and summarized the methodological key details and results (Table 1). Different studies used different DNA replication/detection systems in the gut, ranging from detection of tritiated thymidine incorporation into DNA to various modified nucleosides and cellular fate-mapping using genetic reporter systems with labelling periods ranging from days to several weeks (Table 1).

**Table 1:** Previously Published Research on Neurogenesis in Enteric Nerves After Postnatal Day 21

Mouse strain/genetic background	Age at DNA replication label application	Anatomic region analyzed	Method	No. of ganglia/neurons counted, no. of mice used	ENS neurogenesis/replication after P21	Reference
CD-1	E8-E18, P1-P5, P7, P9, P14, and P21	Duodenum and jejunum	[3H] thymidine; 4 injections in 12 or 24 h, analysis at P30	142 ± 5 myenteric ganglia, 341 ± 42 submucosal ganglia per 1 cm, no. of mice not specified	No	Pham et al, 1991
SvEv129	At least 6 weeks	Small intestine (not specified) and colon	Alzet pump (BrdU ∼30 mg, kg−1, d−1) 7 days, then 2-wk chase	No. of ganglia/neurons not specified, no. of mice not specified	No	Liu et al, 2009
Not specified	E7.5, E8.5, E12.5, P0, P7, P30, P84	Small intestine (not specified)	Lineage tracing (Sox10::iCreERT2;R26eYFP) analysis at P84, P140	More than 3000 neurons, no. of mice = 4	P30 = 1.6% ± 1.1%, P84 = 0.6% ± 0.2%	Laranjeira et al, 2011
C57BL/6J	P120	Stomach, duodenum, distal ileum, cecum, and colon	6-wk pulse, 6-wk chase (intraperitoneal injection of BrdU 50 μg/g body weight, followed by BrdU in the drinking water (0.5 mg/mL) for 6 weeks	More than 1000 neurons per mouse, no. of mice = 3	No	Joseph et al, 2011
C57BL/6J	4 months	Distal colon	EdU (intraperitoneal 50 mg/kg) every 48 hours for 7 days, then every 12 hours for the next 48 hours, and 1 and 2 hours before death	No. of ganglia/neurons not specified, no. of mice = 4	No	Belkind-Gerson et al, 2015
C57BL/6J	2–4 months	Distal colon	Lineage tracing (Sox2CreER:YFP) and EdU (intraperitoneal 50 mg/kg) every 24 hours (total 7 injections)	More than 10,000 neurons to analyze EdU+ neurons, no. of mice = 4	2 mo = 3.5% ± 2.2% YFP+/HuD+ 4 mo = 0.7% ± 1.1% YFP+/HuD+, no EdU+/HuD+	Belkind-Gerson et al, 2017
C57BL/6J	8–24 weeks	Ileum	IdU (1 mg/mL) for 7 days in the drinking water or IdU (1 mg/mL) for 7 days and then exchanged to CldU (1 mg/mL) for 7 days in the drinking water	No. of ganglia/neurons not specified, no. of mice = 3	Yes, ∼70% after 1 week, 88% 2 weeks	Kulkarni et al, 2017
C57BL/6J	8–12 weeks	Ileum and colon	EdU (intraperitoneal 1 mg) for 7 days	Not specified	No	Vicentini et al, 2021

NOTE. Pham et al^4^ analyzed the ENS at P30 after injecting tritiated thymidine 4 times within 24 hours on days P1-P5, P7, P9, P14, and P21, but they reported no enteric neurons in the duodenum and jejunum that had retained the label after P21. Liu et al^5^ did not observe new enteric neurons in the adult mouse gut after continuous application of BrdU for 7 days, followed by a 2-week chase without BrdU. Laranjeira et al^6^ did not find convincing evidence of neurogenesis in the adult small intestine after 1–3 months of age in fate-mapping experiments using *Sox10::iCreER*^*T2*^*;R26ReYFP* mice, which were analyzed for YFP expression at P84 and P130. Joseph et al^7^ did not observe label retention of BrdU in the ENS throughout the GI tract in 4-month-old mice with 6 weeks of BrdU labelling in the drinking water, followed by a 6-week chase without BrdU. Belkind-Gerson et al^8^^,^^9^ used EdU labelling to analyze the distal colon and found no evidence of neuronal replication in adult mice. Similarly, Vicentini et al^10^ found no evidence of neuronal replication after EdU labelling. Kulkarni et al^11^ observed ∼70% turnover rate of enteric neurons after 1 week and 88% turnover rate after 2 weeks using IdU and CldU labelling in the ileum.

Differences in methods (DNA labelling times, antibodies used to detect incorporated nucleotide analogues in the cellular DNA, antigen retrieval methods, and differences in the small intestine region analyzed) all could potentially explain the difference between the results reported by Kulkarni et al^11^ and those by 7 other published studies (Table 1). The study by Kulkarni et al proposed that the failure of other studies to observe ENS proliferation might result from differences in antigen retrieval or from long chase periods, which may lead to a decay of label below detection level. However, a systematic study either addressing these variables or independently replicating the findings from Kulkarni et al is currently missing.

To avoid possible confounding factors associated with comparing previous studies with the results from Kulkarni et al,^11^ we used the same materials and methods described in their study, including the application of the nucleoside analogue 5-iodo-2'-deoxyuridine (IdU) in drinking water with the same concentration and labelling period, DNA denaturation steps, and antibodies. To assess possible anatomic location related variance, we analyzed not only the ileum as in Kulkarni et al but also the duodenum and the jejunum in the small intestine. We carried out analyses in cryosections and whole mount preparations as described in Kulkarni et al. To improve contrast and resolution and to test an independent mounting system, in parallel we also carried out an analysis using paraffin embedding.

Finally, because immunohistochemical detection can be associated with staining artefacts, we also excluded this variable with a parallel analysis using “click chemistry”-based detection of 5-ethynyl-2'-deoxyuridine (EdU), a nucleoside analogue now widely used to detect DNA replication. EdU-based quantification of ENS replication in the small intestine is currently lacking, and published results have been limited to the colon.^8^, ^9^, ^10^

## Results

**Figure 1:**
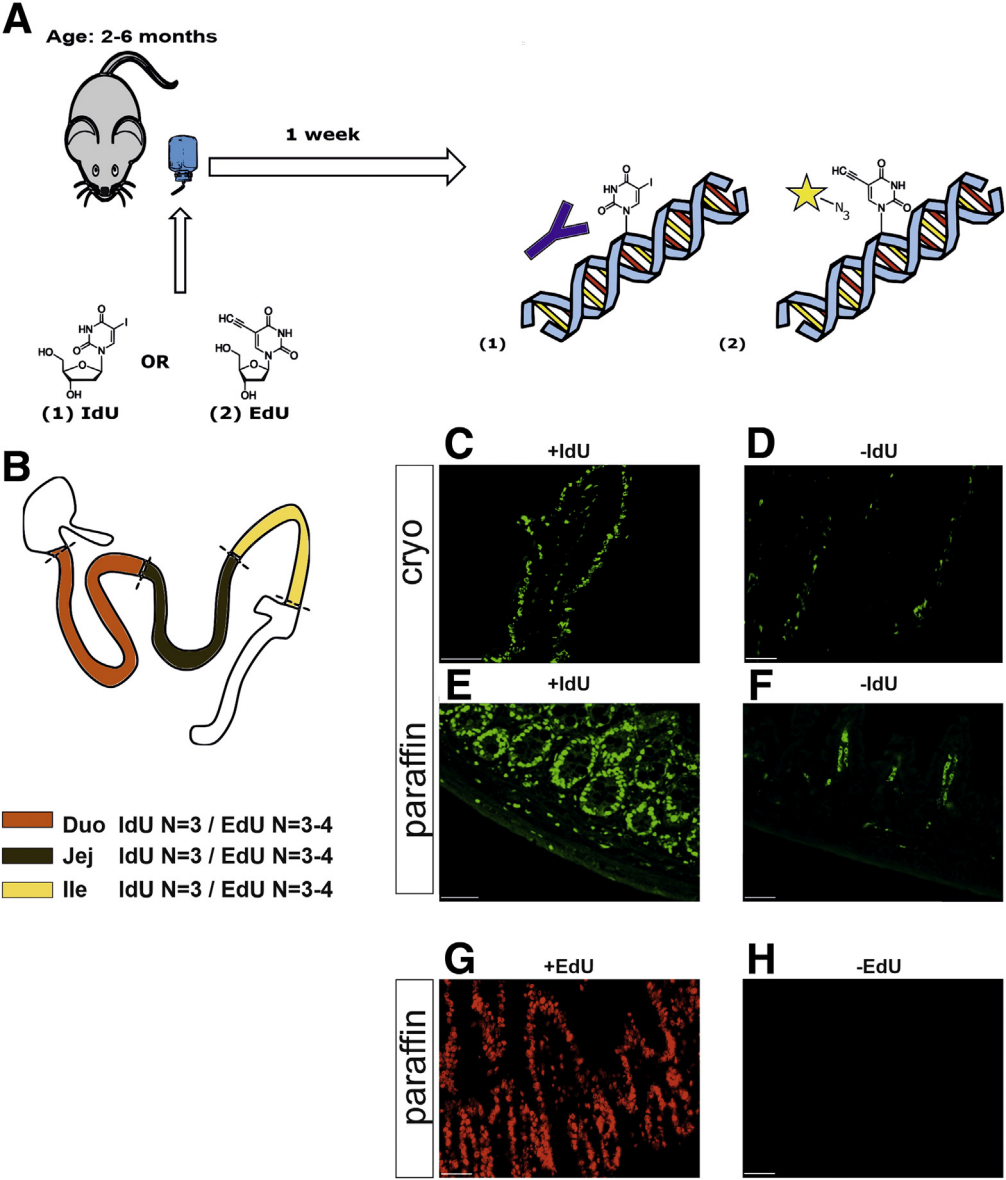
**Study design to analyze ENS proliferation in the small intestine.** (*A*) IdU or EdU was given in drinking water for 1 week, after which the mice were euthanized. (*B*) The small intestine (IdU, N = 3 animals/EdU, N = 3–4 animals) was divided into 3 anatomic segments of about equal length to represent the duodenum, jejunum, and ileum and processed for longitudinal immunohistochemistry using both cryosections and paraffin embedding systems and for LM-MPs for whole mount analysis. (*C*, *E*, and *G*) Epithelial cells show a strong positive signal, demonstrating success of labelling and label detection for both nucleotide analogues. (*D* and *F*) Note background fluorescence in both cryosections and paraffin sections in IdU samples indicating background staining. (*H*) No signal is observed when EdU is omitted, validating specificity. Scale bar, 50 μm.

IdU was administered to adult mice through their drinking water for 1 week as shown in Figure 1*A* and as described by Kulkarni et al.^11^ In parallel, mice were given EdU in drinking water for 1 week (Figure 1*A*). For the quantification of enteric neurons, the small intestine was divided into 3 anatomic segments approximating the duodenum, the jejunum, and the ileum as indicated on Figure 1*B*.

To visualize IdU, we used the same mouse anti–5-bromo-2'-deoxyuridine (BrdU) primary antibody known to cross-react with IdU as was used by Kulkarni et al.^11^ We observed detectable staining in gut samples from mice that did not receive IdU (IdU–), likely reflecting secondary antibody binding to endogenous mouse immunoglobulin G (IgG) as expected in situations when the primary antibody used in the analysis is raised in the same species as the subject of the study (Figure 1*D* and *F*). No background staining was observed after staining with EdU (Figure 1*H*).

**Figure 2:**
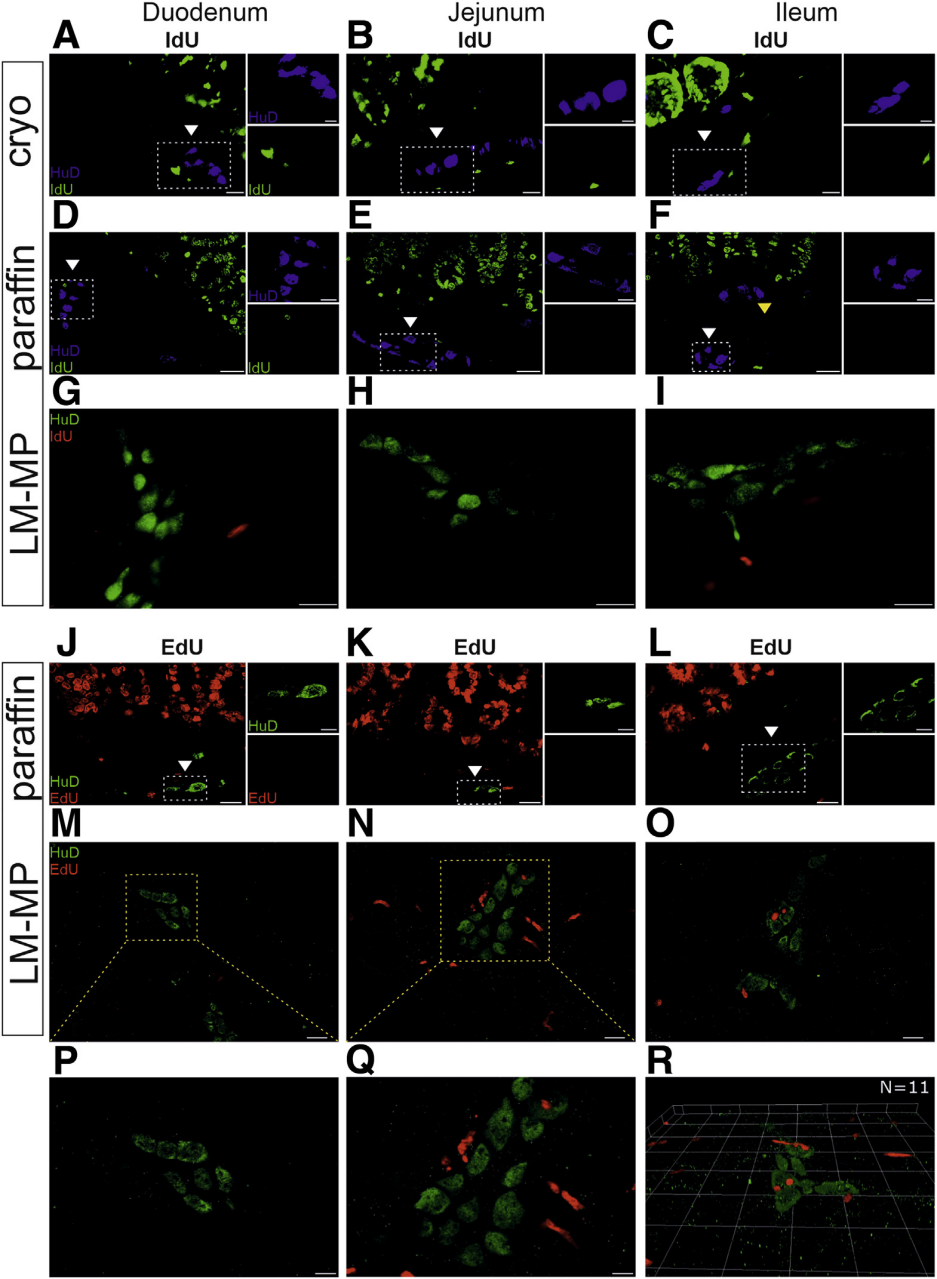
**Analysis of ENS proliferation in the small intestine.** (*A–C*) Longitudinal cryosections and (*D–F*) paraffin sections of the small intestine were immunostained for IdU (*green*) and for HuD (*blue*) to reveal enteric neurons that had undergone replication. Cells in the epithelium show signal for IdU as expected. (*A–F*) Myenteric ganglia are indicated with *white arrowheads* and submucosal ganglia with *yellow arrowheads*. No double-positive HuD+/IdU+ enteric neurons were detected (n = 300 ganglia analyzed in n = 3 animals, age = 24 weeks). *Dashed rectangle* indicates magnified area next to the panel. Scale bar, 20 μm, 10 μm on insets. (*G–I*) Analysis of LM-MPs from IdU-labelled animals revealed no double-positive HuD+/IdU+ enteric neurons (n = 3790 neurons analyzed in n = 3 animals, age = 21 weeks). Scale bar, 20 μm. (*J–L*) Analysis of longitudinal paraffin sections from the small intestine of EdU-labelled animals, HuD (*green*) and EdU (*red*). Cells in the epithelium show signal for EdU as expected. No double-positive HuD+/EdU+ enteric neurons were detected (n = 451 ganglia counted in n = 3 animals, age = 17 weeks). Myenteric ganglia are indicated with *white arrowheads* and submucosal ganglia with *yellow arrowheads*. *Dashed rectangle* indicates magnified area next to the panel. Scale bar, 20 μm, 10 μm on insets. (*M–O*) Analysis of LM-MPs from EdU-labelled animals (n = 1474 neurons analyzed in n = 4 animals, age = 8–10 weeks) revealed no double-positive HuD+/EdU+ enteric neurons. (*P* and *Q*) *Dashed rectangle* indicates magnified images below. (*R*) In 11 neurons in EdU-labelled animals we observed a putative overlap of HuD+/EdU+ labelling. 3D ApoTome analysis revealed that in all cases (n = 11) cells were layered on top of each other along the Z axis, appearing as false positives in 2D microscopy. Scale bar, 20 μm, 10 μm on insets.

Epithelial cells of the gut turn over in less than 1 week and serve as an internal positive control for DNA replication. In line with this, both nucleoside analogues IdU and EdU did label epithelial cells, validating both methods (Figure 1*C*, *E*, and *G*). Microscopic analysis of immunohistochemically labelled small intestine in both cryosections and paraffin-embedded sections, as well as quantification of 300 ganglia in the small intestine, did not reveal IdU-positive (IdU+) enteric nerve cells, whereas the epithelium showed label retention (Figure 2*A–F*, Table 2).

**Table 2:** Summary of the Study Results

Area	Ganglia counted (paraffin sections)		Positive ganglia (paraffin sections)		Neurons counted (LM-MP)		Putative (+) neurons (LM-MP)		3D apotome analysis
	IdU	EdU	IdU	EdU	IdU	EdU	IdU	EdU	EdU
Duodenum	87	140	0	0	1610	555	0	3	Negative
Jejunum	103	153	0	0	928	415	0	4	Negative
Ileum	110	158	0	0	1252	504	0	4	Negative
Total	300	451	0	0	3790	1474	0	0	Negative

NOTE. Quantification of enteric neurons in IdU- and EdU-labelled mice from paraffin sections and LM-MPs are shown from each anatomic segment. IdU paraffin and LM-MP, n = 3 mice; EdU paraffin, n = 3 mice; EdU LM-MP, n = 4 mice.

For a more detailed view of the myenteric plexus, we analyzed longitudinal muscle-myenteric plexus preparations (LM-MPs) throughout the small intestine from mice given IdU. A total of 3790 enteric neurons were analyzed with microscopy (Table 2). Although extraganglionic IdU+ cells were readily observed (Figure 2*G–I*), no IdU+ enteric neurons were detected.

Similarly, analysis of 451 ganglia throughout the small intestine in paraffin-embedded sections using click chemistry–based detection of DNA revealed no EdU+ enteric nerve cells, whereas epithelial cells were readily labelled (Figure 2*J–L*).

Finally, a total of 1474 enteric neurons throughout the small intestine were analyzed with microscopy in the LM-MPs from mice given EdU. Our analysis detected 11 of 1474 cells as potentially double-positive for co-staining of EdU and the neuronal marker HuD. However, 3-dimensional ApoTome analysis revealed that in all 11 cases, EdU and HuD positive cells were not co-stained and were instead stacked on top of each other along the Z axis (Figure 2*R*) and thus did not reflect replication of enteric nerve cells.

## Discussion

Limited in vivo neurogenic potential in the ENS has been observed only after injury^6^^,^^8^, ^9^, ^10^^,^^12^^,^^13^ (Table 1). Kulkarni et al^11^ challenged this dogma, suggesting that almost 70% of myenteric neurons are replaced within 1 week under normal physiological conditions.

Here we replicated the key experiment under the same conditions using the same reagents as used in the study by Kulkarni et al.^11^ Although proliferating epithelial cells were readily detectable, we did not detect enteric neuronal proliferation. Parallel analysis using click chemistry-based DNA labelling that excludes immunohistochemical detection-related variables resulted in the same conclusion. Our results are in line with previously published work (Table 1), which includes incorporation of tritiated thymidine into the DNA, which similarly to click chemistry-based detection of DNA replication excludes artefacts related to immunohistochemical detection.^4^ Because we focused on neuronal replication, we acknowledge that our results do not exclude the possibility of neurogenesis that would occur without cell division. Indeed, enteric neurogenesis through transdifferentiation at less than 1% per week rate has been shown to occur in the mouse gut at 2 and 4 months of age^8^^,^^9^ (Table 1).

Liu et al^5^ showed an increase in relative number of myenteric neurons between 1 and 4 months of age, indicative of postnatal neurogenesis. However, the increase in relative number of myenteric neurons observed by Liu et al occurred at a slow rate because continuous BrdU infusion for 7 days in their study did not reveal enteric neurons that had retained BrdU 2 weeks after chase, which is in agreement with our results.

We conclude that within 1 week in the adult mouse under normal physiological conditions, enteric nerve cells do not replicate.

Although identifying the possible reasons for the observations reported in Kulkarni et al^11^ is not possible for us and remains outside of the scope of the current study, we briefly discuss some factors that may have influenced the result reported in Kulkarni et al.

Because the anti-BrdU antibody is unable to recognize IdU that is incorporated into double-stranded DNA, denaturation of DNA must be carried out to expose single-stranded regions. To do this, Kulkarni et al^11^ used 2N HCl for 5, 10, and 15 minutes at 50°C and saw IdU signal emerging from enteric neurons only after 15 minutes. On the basis of published work, one would expect a time-dependent gradual increase in IdU signal using 2N HCl denaturation,^14^ which Kulkarni et al surprisingly do not observe (S Figure 3 in Kulkarni et al). In the critical IdU and CldU double-labelling experiment that forms the main evidence for the suggested ∼70% ENS turnover in 1 week and 88% turnover in 2 weeks, Kulkarni et al reported a 5- to 10-fold variation between the 3 animals studied (S Table 8 in Kulkarni et al). The lack of a time-dependent gradual increase in IdU signal and major variation between animals may indicate the presence of unnoticed variables. The fact that the repeat of exactly the same experiment with the same reagents and methods did not reproduce the finding, not even partially (Figure 2*A–I*), supports this interpretation and is further supported by the same conclusion using EdU-based click chemistry data (Figure 2*J–R*) and previous studies.^4^, ^5^, ^6^, ^7^, ^8^, ^9^, ^10^ It is also possible that the use of mouse primary antibodies to stain mouse tissue explains some of the results. As our results show (Figure 1*D* and *F*) and as is widely recognized, this can lead to detection of endogenous IgGs with secondary antibody staining. A minor bias may also rise from the lack of 3-dimensional analysis to exclude accidental overlap on the Z-axis.

## Materials and Methods

### Animals

Mice used in this study were of mixed C57BL6/129ola background and of 8–24 weeks of age at the time of analysis. The mice were housed in a 12-hour/12-hour light/dark cycle at 20°C–22°C with ad libitum access to standard chow and water. All animal experiments were approved by the National Animal Experiment Board of Finland.

### IdU and EdU Labelling

Mice were given IdU (1 mg/mL) as described in Kulkarni et al^11^ or EdU (1 mg/mL) for 1 week in drinking water, after which animals were euthanized by lethal exposure to CO₂, followed by cervical dislocation and dissected for analysis.

### Tissue Preparation and Labelling

Cryosections, paraffin-embedded sections, and LM-MP preparations were used for immunohistologic preparations. All samples were fixed in freshly made ice-cold 4% paraformaldehyde in phosphate-buffered saline (PBS) overnight.

### Cryosections

The tissues were stored in phosphate buffer containing 30% sucrose (Thermo Fisher Scientific, Waltham, MA) at 4°C. The 16-μm cryosections were cut using a cryotome (Leica CM 3050S; Wetzlar, Germany). Antigen retrieval was performed in boiling 100 mmol/L citrate buffer pH 6 for 2.5 minutes as described by Kulkarni et al,^11^ followed by washing in PBS thrice (5 minutes per wash). The sections were blocked in 5% normal donkey serum (Abcam, Cambridge, UK) and 0.3% Triton-X100 (Thermo Fisher Scientific) in PBS at room temperature for 1 hour, followed by incubation with a rabbit anti-HuD antibody (1:500 Invitrogen PA5-79199; Waltham, MA) and a mouse anti-BrdU (1:100 B44; Becton, Dickinson and Company Biosciences, Franklin Lakes, NJ) for 48 hours at 4°C in the dark. After washing thrice in PBS (15 minutes per wash), the sections were incubated at room temperature for 1.5 hours with donkey anti-rabbit Alexa 488 (Abcam; Ab150065) and donkey anti-mouse Alexa 594 (Abcam; Ab150112) secondary antibodies. The sections were washed thrice in PBS (15 minutes per wash), and a coverslip was mounted using mounting medium (Thermo Fisher Scientific; Shandon Immu-Mount).

### LM-MP

The small intestine was cut into 2-cm-long segments, and LM-MPs were peeled off from the intestinal tissue by microdissection. The tissues were permeabilized with 1% Triton-X100 (Thermo Fisher Scientific) for 1 hour. In IdU-labelled mice before label detection, antigen retrieval was performed in 2 N HCl at 50°C for 15 minutes, followed by three 15-minute washes in PBS as described by Kulkarni et al.^11^ In EdU-labelled mice, detection of EdU was done using a Click-iT EdU cell proliferation kit (Invitrogen C10339) according to the manufacturer’s instructions. The Click-iT reaction cocktail required for detection was prepared immediately before use. The samples were incubated in the Click-iT reaction cocktail mix for 1 hour in the dark and then washed thrice with PBS (5 minutes per wash). Next, the tissues were blocked in 5% normal donkey serum (Abcam) and 0.3% Triton-X100 (Thermo Fisher Scientific) in PBS for 1 hour at room temperature. In IdU-labelled mice, the tissue was then incubated with a rabbit anti-HuD antibody (1:500 Invitrogen PA5-79199) and a mouse anti-BrdU antibody (1:100 B44; Becton, Dickinson and Company Biosciences) for 48 hours at 16°C in the dark as described by Kulkarni et al. In EdU-labelled mice, blocking was followed by incubation with a rabbit anti-HuD antibody (1:500 Invitrogen PA5-79199) overnight at 4°C in the dark. After washing thrice in PBS (15 minutes per wash), the tissues were incubated for 1 hour at room temperature with donkey anti-rabbit Alexa 488 (Abcam; Ab150065) and for anti-BrdU detection with donkey anti-mouse Alexa 594 (Abcam; Ab150112) secondary antibodies. Finally, the tissues were washed thrice in PBS (15 minutes per wash), and a coverslip was placed on the slide using mounting medium (Thermo Fischer Scientific; Shandon Immu-Mount).

### Paraffin-Embedded Sections

The tissues were dehydrated and embedded using an ASP300 S tissue processor (Leica). The 5-μm longitudinal paraffin sections were cut using a Tissue-Tek microtome (Sakura, Osaka, Japan). The sections underwent deparaffinization through serial washes with xylene, alcohol, and water.

For IdU-labelled mice, antigen retrieval was performed in 2 N HCl at 50°C for 15 minutes, followed by three 15-minute washes in tris-buffered saline tween (TBS-T). In EdU-labelled mice, after 10-minute antigen retrieval with boiling 10 mmol/L citrate buffer pH 6 and subsequent washes, detection of EdU was done according to the manufacturer’s instructions as described earlier.

The sections were blocked in 5% normal donkey serum (Abcam) in tris-buffered saline containing 0.1% Tween-20 (Thermo Fischer Scientific) for 30 minutes at room temperature and then incubated with a rabbit anti-HuD antibody (1:500 Invitrogen PA5-79199), and for IdU-labelled sections, a mouse anti-BrdU (1:100 B44 Becton; Dickinson and Company Biosciences) was used for 24–48 hours at 4°C in the dark. After three 15-minute washes in TBS-T, the sections were incubated for 1.5 hours at room temperature with donkey anti-rabbit Alexa 488 (Abcam; Ab150065) and donkey anti-mouse Alexa 594 (Abcam; Ab150112) secondary antibodies. The sections were washed thrice in TBS-T (15 minutes per wash), and coverslips were mounted using mounting medium (Thermo Fischer Scientific; Shandon Immu-Mount).

### Imaging

Slides were imaged with a Zeiss AxioImager (Oberkochen, Germany) microscope outfitted with a Zeiss 3D ApoTome for optical sectioning. Both the longitudinal sections containing enteric ganglia and LMMPs were imaged at several different magnifications to identify possible co-localization with HuD and IdU or EdU. Potential co-localization was investigated using the 3D ApoTome optical sectioning with at least 5 successive fluorescence images with structured illumination to produce a 3-dimensional image.

## Quantitation

Longitudinal sections containing the whole small intestine were used. In total, 751 ganglia in IdU-labelled and EdU-labelled mice were assessed from the duodenum, jejunum, and ileum to detect proliferation in the enteric ganglia. LM-MPs from the duodenum, jejunum, and ileum of mice were used. A total of 5264 enteric neurons were counted to look for potential individual neurons that had retained IdU or EdU.
